# Conference Report: ACSICON 2008

**Published:** 2009

**Authors:** Narendra Patwardhan

**Affiliations:** *Consultant Dermatologist, Shreeyash Hospital, Pune, Maharashtra, India*

The eighth biennial conference of ACSI held in the beautiful city of Indore from 21-23 November '08 was organized by the Ujjain dermatology association at Devi Ahilya University Cultural Centre. Indore is the largest city in Madhya Pradesh and is close to the pilgrimage town of Ujjain. It was attended by an impressive 400 delegates. The theme of the conference was therapy of pigmentary disorders.

The first day (23 November 2008) started with a session on the basics of dermatosurgery. In a lucid presentation, Dr. Sharad Mutalik insisted on proper informed consent, lignocaine hypersensitivity testing and preoperative photographs. Various topical anesthetic agents, nerve and regional blocks were highlighted. Medical emergencies during surgery were elaborated by Dr. Yashwant Tawade. He highlighted the need for maintenance of emergency trolley which should include subcutaneous adrenaline, Ambu bag. Differentiation between true lignocaine hypersensitivity and vasovagal syncope was also discussed.

Dr. Sanjay Kucheria, plastic surgeon from Indore, stressed on the need for suturing in two layers along Blaschko's lines, to ensure cosmetically acceptable scar on the face. For young dermatosurgeons, setting up a proper dermatosurgery theatre with appropriate use of available space was highlighted in an excellent lecture by Dr. Dhanashree Bhide.

An entire session was devoted to vitiligo surgery, a disease which is of great significance in India. Dr. Niti Khunger, President ACSI, discussed surgical management of difficult areas like lips, nipples, genitalia and acral areas in a masterly presentation. She insisted on deep dermabrasion for palms and soles, split thickness skin grafting STSG for entire nipple and areola, and the need for proper immobilization by use of splints for joint areas.

Autologous non-cultured epidermal cell suspension has been receiving great interest of late and this method was discussed by Dr. Malhotra for extensive stable areas of vitiligo. An innovative technique called smash grafting was presented by Dr. KG Singh.

Dr. Somesh Gupta showed excellent results by punch grafting by using small punch grafts (1-2 mm), which help to avoid cobblestoning.

Dr. Uday Khopkar discussed the role of medical management in children. Oral and topical steroids for short periods to halt progress of vitiligo, followed by NBUVB for management was described as a safe and effective method in children. Dr. Munish Paul talked about targeted phototherapy, which is particularly suited for children.

In the afternoon session on lasers, Dr. Sanjeev Aurangabadkar talked about the current status of management of pigmented lesions like café-au-lait macules, lentigenes, tattoos, with Q-switched Nd-YAG laser. Despite the recent advances in technology, multiple sittings (5-20) are necessary. Dr. Pradyumna Vaidya gave an elaborative talk on the use of CO_2_ laser for partial matricetomy.

As the theme of the conference was pigmentary disorders, the showpiece of the conference was the management of melasma.

Dr. KS Dhillon in his talk elaborated on the role of various medications like azelaic acid, kojic acid, hydroquinone, arbutin, paper mulberry, and also the role of lasers, intense pulsed light (IPL), fractional photothermolysis in the management of melasma with their pros and cons. Dr. Sangeeta Amladi discussed various chemical peels like salicylic acid 25%, TCA 10%, and glycolic acid 50-70%. Dr. Mukta Sachdev, Dr. Upadhayay, and Dr. Jayashree Sharad talked about the etiopathogenesis and use of lactic acid and arginine peels in perioral and periorbital melanosis. Dr. Penpun from Bangkok discussed her experience with latest Q-switched Nd-YAG and its cost-effectiveness.

Overview of vascular lesions and lasers was presented by Dr. Narendra Patwardhan. The first day ended with a talk on the management of keloids by veteran dermatosurgeon Dr. Satish Sawant.

The second day was workshop day. The team was led by Dr. Dilip Hemnani. The entire spectrum of cutaneous procedures was demonstrated, including radiofrequency, microdermabrasion, cryosurgery, sclerotherapy, dermaroller and innovative methods in vitiligo management by Dr. Dilip Kachawa, Dr. Somesh Gupta, Dr. Dilip Shah, Dr. Ashok Parakh, Dr. KG Singh, Dr. Chandrashekhar and others. Advanced surgical procedures such as liposuction and hair transplantation were explained with very lucid videos by Dr. Venkataram Mysore, Shrirang Pandit and Dr. Narendra Patwardhan. International faculty, Dr. Eric, Dr. Penpun and Dr. Won Soon Chung demonstrated the use of lasers for leg veins, pigmented lesions, IPL and radiofrequency RF for skin rejuvenation. In the afternoon session, Erbium glass laser, fractional CO_2_, and skin tightening of the face were demonstrated by Dr. MK Shetty, Dr. Minal Patwardhan, Dr. Munish Paul, Dr. Amladi, and Dr. Upadhaya.

The third and last day started with the free paper session, followed by a panel discussion on hot topics like hair removal lasers, scar management, new lasers etc. ACSICON '08 was thus a very successful conference. All talks were followed by detailed question and answer sessions which were elaborative and educational.

The auditorium was excellent with state-of-the-art audiovisual facilities. Hosting such conferences in smaller cities such as Indore will help create awareness about cutaneous surgery and give a fillip to the subspeciality. Dr. K Mishra, Dr. Hussain, Dr. Hemnani and their local team deserve full praise and appreciation.

The next workshop will be held in Srinagar, on in October 2009 and in 2010, ACSICON 2010 will be held in Aurangabad in October-November 2010 under leadership of Dr. Aniruddha Gulanikar.

